# Anopheline mosquito saliva contains bacteria that are transferred to a mammalian host through blood feeding

**DOI:** 10.3389/fmicb.2023.1157613

**Published:** 2023-07-18

**Authors:** Anastasia Accoti, Claudia Damiani, Emilia Nunzi, Alessia Cappelli, Gloria Iacomelli, Giulia Monacchia, Antonella Turco, Francesco D’Alò, Matthew J. Peirce, Guido Favia, Roberta Spaccapelo

**Affiliations:** ^1^Department of Medicine and Surgery, CIRM Italian Malaria Network Perugia, Functional Genomic Center (C.U.R.Ge.F), University of Perugia, Perugia, Italy; ^2^School of Biosciences and Veterinary Medicine, University of Camerino, CIRM Italian Malaria Network, Via Gentile III da Varano, Camerino, Italy; ^3^Interuniversity Consortium for Biotechnology (C.I.B.), Trieste, Italy

**Keywords:** mosquito, saliva, microbiota, *Serratia*, *plasmodium*

## Abstract

**Introduction:**

Malaria transmission occurs when *Plasmodium* sporozoites are transferred from the salivary glands of anopheline mosquitoes to a human host through the injection of saliva. The need for better understanding, as well as novel modes of inhibiting, this key event in transmission has driven intense study of the protein and miRNA content of saliva. Until now the possibility that mosquito saliva may also contain bacteria has remained an open question despite the well documented presence of a rich microbiome in salivary glands.

**Methods:**

Using both 16S rRNA sequencing and MALDI-TOF approaches, we characterized the composition of the saliva microbiome of *An. gambiae* and *An. stephensi* mosquitoes which respectively represent two of the most important vectors for the major malaria-causing parasites *P. falciparum* and *P. vivax*.

**Results:**

To eliminate the possible detection of non-mosquito-derived bacteria, we used a transgenic, fluorescent strain of one of the identified bacteria, *Serratiamarcescens*, to infect mosquitoes and detect its presence in mosquito salivary glands as well as its transfer to, and colonization of, mammalian host tissues following a mosquito bite. We also showed that *Plasmodium* infection modified the mosquito microbiota, increasing the presence of Serratia while diminishing the presence of *Elizabethkingia* and that both *P. berghei* and *Serratia* were transferred to, and colonized mammalian tissues.

**Discussion:**

These data thus document the presence of bacteria in mosquito saliva, their transfer to, and growth in a mammalian host as well as possible interactions with *Plasmodium* transmission. Together they raise the possible role of mosquitoes as vectors of bacterial infection and the utility of commensal mosquito bacteria for the development of transmission-blocking strategies within a mammalian host.

## Introduction

The previous two decades saw striking reductions in malaria morbidity and mortality in endemic countries but in recent years this promising progress, has stalled (WHO, 2021). Vector-based strategies, insecticide-treated bed nets (ITNs) and indoor residual spraying (IRS), have proved highly effective in reducing transmission (Delves et al., 2018) but are now beset by difficulties as resistance to their aging active ingredients spreads among vector populations (Ranson and Lissenden, 2016). At the same time, the *Plasmodium* parasites that cause the disease are also developing resistance to the most widely used therapies, including artemisinin (Balikagala et al., 2021). This has led to a renewed imperative to design novel strategies to diminish the parasite-transmitting capacity of the mosquito (Caragata et al., 2020).

One powerful approach aims to use the bacteria that inhabit the mosquito to erect physiological or immunological barriers to infection, either through resource competition, physical interference or immune priming, such that the mosquito becomes refractory to *Plasmodium* infection (Cirimotich et al., 2011a,b; Capone et al., 2013; Guégan et al., 2018). Symbiotic bacteria represent an appealing target since they inhabit the same organs through which the parasite passes as it establishes infection in the mosquito (midgut) and, later, as it is transmitted to a new mammalian host (salivary glands) (Wang et al., 2011; Sharma et al., 2014; Tchioffo et al., 2015; Mancini et al., 2018). In addition, some symbiotic bacteria also have the capacity to invade reproductive organs and thus allow bacteria in a female to be transferred to her off-spring (Werren et al., 2008; Damiani et al., 2010; Wang et al., 2011; Perlmutter and Bordenstein, 2020). One widely cited example is the natural endosymbiont, *Wolbachia*, which has been successfully deployed to block transmission of arboviruses that cause Dengue Fever, Chikungunya and Zika mediated by *Aedes* mosquitoes (Zheng et al., 2019; Caputo et al., 2020; Crawford et al., 2020). While natural populations of *Wolbachia*, and other commensal bacteria (Cirimotich et al., 2011a), are able to block pathogen transmission, an alternative or complementary approach is to genetically modify a symbiotic bacterium such that it expresses an exogenous gene to block infection of a pathogen: paratransgenesis (Durvasula et al., 1997).

Encouraging recent data indicates the presence of stable and high frequency *Wolbachia* infections in some wild Anophelines (Walker et al., 2021). However, the possible exploitation of these bacteria as a useful tool for vector control in the most important African vectors of malaria such as *Anopheles (An.) gambiae* and *An. coluzzii,* remains to be demonstrated. However, other bacteria offer great promise. One example of a mosquito symbiont is *Serratia marcescens* (*S. marcescens*), a gram negative bacterium isolated from anopheline ovaries that can be transferred from one generation to the next (Wang et al., 2017). A genetically modified version of this bacterium renders infected females refractory to *Plasmodium* infection (Wang et al., 2012) while a naturally occurring variant of the same bacterial genus, *S. ureilytica*, has been found to secrete an anti-plasmodial lipase with similar transmission blocking activity (Gao et al., 2021). Beyond the ability of particular symbionts to render mosquitoes more resistant to infection, other aspects of mosquito biology, such as reproductive success and longevity, that are pivotal to vector capacity, can also be influenced by alterations in the mosquito microbiome (Dennison et al., 2016; Cansado-Utrilla et al., 2021). This awareness has driven explosive growth in the characterization of the microbiomes of an array of different anophelines (Gimonneau et al., 2014; Mancini et al., 2018), their life stages and tissues (Tchioffo et al., 2015; Mancini et al., 2018).

One site at which the microbiome remains to be defined is the saliva itself. In *An. gambiae* mosquitoes *Rickettsia felis* bacteria, acquired through feeding on an infected mouse, were subsequently detected in the blood of mice on which infected mosquitoes had fed. These data are consistent with the possibility of saliva-mediated transmission (Dieme et al., 2015) but the presence of bacteria in the saliva was not directly tested.

In contrast, the protein content of saliva (Arcà et al., 2007; Ribeiro et al., 2010; Scarpassa et al., 2019) has been intensively studied and revealed to play a role in acute inflammatory, complement and clotting responses in the human host (Ribeiro et al., 2010; Fontaine et al., 2011; Arcà and Ribeiro, 2018; Arcà et al., 2019). Moreover, antibody responses to some of these protein components have been detected and proposed as biomarkers for recent mosquito contact (Rizzo et al., 2011, 2014). Saliva may also modulate the character of the adaptive immune response that develops to an invading pathogen, favoring (Schneider and Higgs, 2008) or hindering (Donovan et al., 2007) its transmission. Moreover, some saliva components appear to impact directly on sporozoite function, for example inhibiting their capacity to invade cells, thus limiting transmission (Schleicher et al., 2018), or physically protecting them from immune destruction by the host, thereby promoting transmission (Arora et al., 2021).

Studies using intravital imaging have visualized the ‘first contact’ between *Plasmodium*, along with the saliva in which it is delivered, and the mammalian immune system. Only a few hours after being deposited under the skin during an infected mosquito bite, sporozoites can be detected in draining lymph nodes, interacting with dendritic cells, the specialized antigen-presenting cells that initiate adaptive immune responses (Amino et al., 2006). Together, these findings highlight the extent to which the contents of the saliva interact directly with the parasite as well as with the host immune system, to modulate anti-plasmodial immunity, and thus the success or otherwise of *Plasmodium* infection (Fontaine et al., 2011; Arcà and Ribeiro, 2018).

This understanding has been exploited to develop innovative transmission blocking strategies. For example, the ‘flying vaccinator’ concept aims to utilise transgenic mosquitoes as vehicles for vaccine delivery (Yamamoto et al., 2010). Mosquitoes would be engineered to express vaccine candidates in their saliva which are then delivered through blood feeding on a mammalian host. Significant practical and ethical obstacles make deployment of this idea unlikely in humans; the lack of control of the amount of ‘vaccine’ delivered to each individual and the lack of informed consent have been sited (Crampton, 1999). However, proof of principal studies have demonstrated its feasibility (Yamamoto et al., 2012; Sumitani et al., 2013) and its application to the vaccination of wild animals remains a possibility.

In this study we set out to test the possibility that, given the rich microbiome previously described in the salivary glands (Sharma et al., 2014; Tchioffo et al., 2015; Mancini et al., 2018), the saliva itself might also contain bacteria. Using *An.gambiae* and *An. stephensi*, primary vectors for two of the most important *Plasmodium* (*P.*) species for human transmission, *P. falciparum* and *P. vivax* respectively, we showed that not only does saliva contain bacteria, but these bacteria are transferred to and are able to colonize tissues within the mammalian host following a blood meal. The possible implications of these findings for future transmission-blocking strategies are discussed.

## Materials and methods

### Ethics statement

Experiments with animals performed were carried out in accordance with the EU Animals Act 1986. Mosquito infections with *Plasmodium berghei* (*P. berghei*) by blood feeding on parasitized mice were approved by the Italian Ministry of Health and carried out under the License 1184/2020-PR based on the D.lgs. 26/2014. All the procedures were of mild-to-moderate severity, and protocols were designed to minimize the numbers of animals used. Opportunities for reduction, refinement, and replacement of animal procedures were constantly reviewed and considered.

### Mosquito rearing

*M*osquito strains were reared in a containment level 2 facility at the department of Medicine and Surgery, Perugia, Italy, authorization N. PG/IC/Imp2/13/001-Rev2 from the Ministry of Health. *An. gambiae,* G3 strain (MR4, MRA-112) and *An. stephensi* (Sind-Kasur Nijmegen strain) (Feldmann and Ponnudurai, 1989) were reared at ~28°C and 60–80% humidity and exposed to a 12/12 h day/night cycle. The larvae were raised following the MR4 protocol^1^ and adults were fed on 6% glucose containing the anti-fungal agent methylparaben (0.1%) (Sigma-Aldrich). Mosquito feeding was performed weekly using a Hemotek PS5 membrane feeder system (Discovery Workshops, United Kingdom) with bull’s blood.

### Mosquito infection with *Plasmodium berghei* parasites and sample preparation for metagenomic analysis

*P. berghei* parasites 662 cl1 line^2^ or GFP-expressing *P. berghei* parasites (Bergreen) (Matz et al., 2013) 5×10^6^ were injected intraperitoneally into CD1 mice (Charles River). Three days later, parasitemia was assessed by Giemsa staining (Hemacolor Rapid staining: Merck), and the presence of gametocytes (0.8–1.5%) was verified. *An. gambiae* and *An. stephensi* females (3–4 days old) were allowed to feed for 20–30 min on anesthetized uninfected or *P. berghei*-infected mouse. Mice were anesthetized intraperitoneally with ketamine and xylazine 100 mg/kg and 40 mg/kg, respectively. Midguts (10 per group) were collected at day 14 post blood feeding (PBF) and *P. berghei* infection evaluated by the presence of fluorescent oocysts (Evos FL Imaging System AMG). At day 21 PBF salivary glands and saliva (35 and 50 mosquitoes per group, respectively) were collected. For *P. berghei*-infected groups, sporozoites in the salivary glands were counted in a Neubauer haemocytometry chamber by optical microscopy. Mosquito saliva was collected by placing mosquitoes around wells of a glass microscope slide containing 50 μL of sterile filtered 6% glucose solution and left to feed for 20 min. As a negative control, 50 μL of the same glucose solution was recovered from a glass slide without mosquito exposure. Sugar feeding was verified by stereomicroscopic examination of mosquito abdomen. Before dissection, commensal surface bacteria on the carcass were removed by washing the mosquito four times with absolute ethanol under a laminar flow hood.

### 16S ribosomal RNA gene sequencing

DNA for metagenomic analysis was isolated from the above samples using a DNeasy Blood & Tissue Kit (Qiagen) according to the manufacturer’s instructions. The microbiota composition was evaluated by 16S SSU rRNA. Sequencing libraries were prepared using a NEXTERA XT DNA sample preparation kit (Illumina) according to the manufacturer’s instructions. The V4 region of the bacterial 16S genes was amplified using primers 515F and 806R (TCGTCG GCAGCGTCAGATGTGTATAAGAGACAGGTGCCAG CMGCCGCGGTAA and GTCTCGTGGGCTCGGAGATGTGTAT AAGAGACAGGGACTACHVGGGTWTCTAAT, respectively) and sequenced using the MiSeq platform (Illumina), by Polo GGB Srl (Siena, Italy).

### Bioinformatic analysis

Demultiplexing of all libraries for each sequencing was accomplished by the Illumina bcl2fastq 2.17.1.14 software. Only reads with at least 100 nucleotides were retained and then primer sequences were detected, clipped and oriented into forward-reverse primer orientation, and analyzed by using the QIIME2 platform (Bolyen et al., 2019) in a genomic cloud -computing environment based on (Felicetti et al., 2014) and designed for biological nano-communication systems in blood vessels for early tumor medical diagnosis (Reali et al., 2018). As a first step, paired-end sequences were denoised, dereplicated, filtered by both any phiX reads and chimera (consensus), by using the q2-dada2 quality control method (Callahan et al., 2016). In particular, the q2-dada2 method makes use of sequence error profiles to obtain putative error-free sequences, referred to as sequence variants (SVs). SVs were assigned taxonomy using a Naive Bayes classifier model trained on the Silva138 99% database trimmed to the V4 region of the 16S. The classifier was then applied to the obtained SVs for mapping them to taxonomy. 16S SVs were agglomerated into Phylum, Class, Order, Family, Genus and Species levels within QIIME2 for evaluating the corresponding taxonomic abundance, alpha and beta diversity analysis. Sequenced samples were provided as ASV abundance tables (rarefied at 8000 reads).

### Mosquito dissection and sample preparation for MALDI-TOF analysis

Tissues (midguts, salivary glands and saliva) were collected under sterile conditions from 3–4 day old, blood-fed *An. gambiae* and *An. stephensi* females as described above. Fresh organs were homogenized in 50 μL of sterile PBS 1X and bacteria grown in enrichment medium, Tryptic Soy (TS) broth, at 37°C and at 120 rpm. The bacterial suspension was sub-cultured on various enrichment agar plates, TS, MacConkey (MCK), Muller Hinton (MH) and Columbia (CNA). At least three individual colonies for each sample were analyzed by MALDI-TOF MS (Bruker). The peptide mass fingerprint (PMF) profile was analyzed using Biotyper 3.0 software (Bruker), and default parameter settings on the commercial Bruker database. Scores of ≥2.0 were considered high confidence (acceptable species-level identification).

### *Serratia* engineering

*Serratia marcescens* were transformed as described elsewhere (Sakural and Komatsubara, 1996). Briefly, *An. gambiae* salivary gland-derived *Serratia* cells which are susceptible to gentamicin but resistant to many other antibiotics, were grown overnight in antibiotic-free Luria Bertani medium (LB) broth at 30°C. The culture was diluted 1:20 into 50 mL of LB and allowed to reach early log phase (optical density at 600 nm, 0.6–0.7). Bacteria were then recovered by centrifugation, resuspended and washed twice with ice-cold sterile 1x PBS containing 10% glycerol. Competent cells (2×10^11^ in 100 μL) were electroporated (12.5 kV cm^−1^, 25 μF, 800 Ω; Bio-Rad Gene Pulser) in a cooled 0.2-cm cuvette with pEP933 plasmid DNA containing DsRed, obtained from *Serratia* Db11dsRED, as well as both tetracycline- and gentamicin-resistance cassettes, a generous gift from Dr. D. Ferrandon (Nehme et al., 2007). LB broth was immediately added to the cuvette and an aliquot plated on LB agar plates supplemented with with 20 μg/mL gentamicin. After 72 h at 30°C, gentamicin-resistant transformants were analyzed by fluorescence microscopy. The isolated colony, henceforth referred to as *Serr^AgDsRed^,* was cultured in gentamicin to maintain extra-chromosomal expression of the plasmid and its identity confirmed by Sanger sequencing (Eurofins Genomics) of PCR-amplified 16S rRNA gene using the universal bacterial primers 27F (AGAGTTTGATCATGGCTCAG) and 1492R (TACGGYTACCTTGTTACGACTT).

### *Serratia^AgDsRed^* mosquito colonization and survival

*Serratia^AgDsRed^* bacteria were cultured to log phase (OD 0.6–0.7) as described above, then maintained at RT (25°C) for 48 h to allow the bacteria to express DsRed protein. Bacteria were harvested, washed twice in sterile PBS 1X and resuspended in 25 mL of sterile 6% glucose solution at a concentration of 10^9^ cells/ mL. Mosquitoes were allowed to feed on *Serratia^AgDsRed^*-glucose solution for 4 days and then on 6% glucose supplemented with gentamicin (20 μg/mL) for the entire duration of the experiment. Mosquito tissues infected with *Serr^AgDsRed^* were analyzed with a Nikon C2^+^ Laser Scanning Confocal microscope at 4, 10, 15, 20 days post bacterial sugar feeding. A total of 50 midguts, salivary glands and ovaries were collected at each time point, in three independent experiments. For mosquito survival the daily mortality rate of 150 *Serr^AgDsRed^* -exposed or unexposed females was monitored for 20 days. To confirm *Serr^AgDsred^* colonization, 4 days after the bacterial challenge, 10 mosquito midguts were analyzed by fluorescence microscopy. For both mosquito species three independent experiments were performed.

### Release of *Serratia^AgDsRed^* during blood feeding on mouse

The transmission of *Serr^AgDsRed^* to a mammalian host during blood feeding was assessed by allowing *Serr^AgDsRed^-*exposed *An. gambiae* and *An. stephensi* females (maximum 15 mosquitoes) to feed on mouse ears or bellies. Mice were anesthetized intraperitoneally with ketamine and xylazine 100 mg/kg and 40 mg/kg, respectively. Bites were confirmed by the appearance of a small hematoma on the mouse ear. After euthanasia, by neck dislocation with the animal under deep anesthesia, ears were washed in Ethanol 70% and sterile 1X PBS. The transmission of *Serr^AgDsRed^* to the ears was confirmed through: (i) DsRed gene amplification using DsRed-specific primers, (Fwd. GGAGTTCA TGCGCTTCA and Rev. GGACAGCTTCTTGTAGTCGGGG), using Amplitaq Gold 360 polymerase (Thermofisher) according to the manufacturer’s instructions; (ii) culture on LB medium containing 20 μg/mL gentamicin; and (iii) confocal microscopic examination of longitudinal ear sections.

A maximum 15 mosquitoes were allowed to feed on mouse belly, then 24 and 48 h after blood feeding, liver, lung, kidney, spleen, brain and heart were recovered from anesthetized mice (CD1 and NOD *scid* gamma), homogenized, and bacteria grown in LB medium or plated on LB agar with gentamicin (20 μg /ml) and grown at 30°C for 48 h. As a positive control three CD1 mice were injected intraperitoneally with 200 μL of 10^8^/ml suspension of *Serr^AgDsRed^*. As a negative control mouse ears and tissues were collected from mice exposed to non-*Serr^AgDsRed^* -infected mosquitoes.

### Mosquito co-infection with *Plasmodium berghei* parasites and Serratia^AgDsRed^

*An. gambiae* and *An. stephensi* mosquitoes were allowed to feed on *Serratia^AgDsRed^*-glucose solution for 5 days and then blood fed on a *P. berghei* infected-mouse as described above. 14 days post *P. berghei* infection 10 midguts were dissected to check for the presence of *Serratia^AgDsRed^* and Bergreen parasites. Twenty-one days post *P. berghei* infection 15 mosquitoes were allowed to feed on a CD1 mouse. Forty-eight hours post infection mice were sacrificed and liver collected for DNA extraction. DsRed was amplified using primers described above and GFP with primers eGFP Fwd. GTGACCACCCTGACCTAC and eGFP Rev. CCATGATATAGACGTTGTGGCTGT.

### Statistical analysis

Differences in the microbial communities identified from several organs of the same mosquito species, or from the same organ under different treatments (*P. berghei*-infected or uninfected), were analyzed in Qiime and scored using the Shannon and Chao1 diversity indexes. Differences in those scores were compared using Kruskal-Wallis tests between groups. Beta diversity of the same bacterial populations was assessed using principal component analysis (PCA). Differences in the infection rate of organs with *Serratia^DsRed^* over time were compared using a Fisher’s exact test while differences in survival rate were evaluated using a log-rank (Mantel-Cox) test both using GraphPad PRISM 9 software.

### Data availability

The data for this study have been deposited in the European Nucleotide Archive (ENA) at EMBL-EBI under accession number PRJEB63521 (https://www.ebi.ac.uk/ena/browser/view/PRJEB63521).

The data and the materials of this study are available to interested researchers, upon request to the corresponding author.

## Results

### Anopheline mosquito saliva contains bacteria

**Figure 1:**
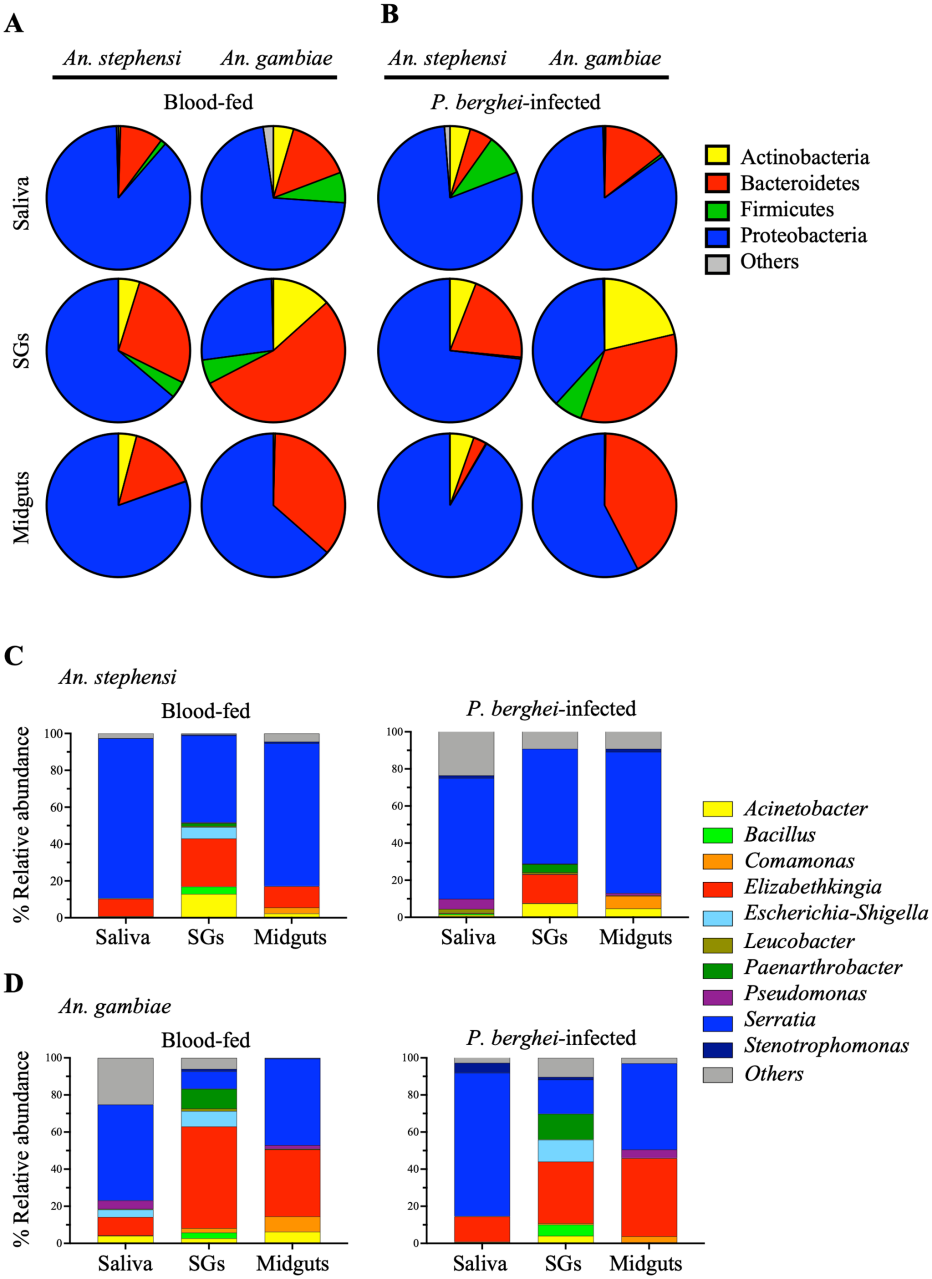
Relative abundance of the most abundant bacteria in different organs (saliva, salivary glands (SGs) and midguts) of female *An. stephensi* and *An. gambiae* mosquitoes in the presence or absence of *P. berghei* infection. Bacterial profiles at the phylum **(A,B)** and genus **(C,D)** level identified in mosquito midguts collected at day 14 post blood feeding and saliva and salivary glands collected at 21 days post blood feeding on *P. berghei*-infected or uninfected mice. Bacteria with a relative abundance lower than 0.4% or not identified were grouped together in ‘Other’. The values represent of the median of three independent experiments.

We first attempted to document the presence of bacteria in the saliva of two anophelines: *An. stephensi* and *An. gambiae*, primary vectors for the human *Plasmodium* parasites, *P. vivax* and *P. falciparum,* respectively. As a reference, salivary glands and midguts, recovered from the same cohort of mosquitoes, were analyzed in parallel. Moreover, to examine the impact of *Plasmodium* infection on the microbiome, organs recovered from females blood-fed on a *P. berghei*-infected mouse were compared with those of females who had fed on an uninfected mouse. Organs were harvested at 14 days post blood feeding (PBF, midguts) or 21 days PBF (salivary glands and saliva). Using 16S rRNA gene sequencing we analyzed 44 samples and a total of 19,116,213 reads were generated (Supplementary Tables S1, S2). After quality filtering and the removal of chimeric sequences, 15,927,132 reads were assigned to amplicon sequence variants (ASVs) at 99% identity and on average, there were 353,936 reads per mosquito sample (Supplementary Table S2). Following taxonomic analysis of ASVs at the phylum level only taxa that had an overall abundance equal to or greater than 0.4% (Supplementary Table S3, S4) were included. The distribution of bacterial taxa among the samples at the phylum (Figures 1A,B) and genus levels (Figures 1C,D) and their relative abundance in the saliva and tissues (Supplementary Tables S4, S5) of uninfected or *P.berghei*-infected *An. gambiae* or *An. stephensi* are reported.

Most strikingly, after an uninfected blood meal the saliva of both anophelines was abundantly populated with bacteria, predominantly those of the phyla *Proteobacteria* and *Bacteroidetes*. In *An. stephensi* the relative abundance of the bacterial populations detected in saliva essentially mirrored those observed in the salivary glands and in the midgut of the same mosquitoes identifying the *Proteobacteria* as the main phylum (88.4%, 63.8% and 80.4% respectively, see Supplementary Table S4). The saliva also contains 9.7% of *Bacteroidetes* and low levels of *Firmicutes* and *Actinobacteria* (1.2% and 0.5% respectively) (Figure 1A and Supplementary Table S4). In contrast, in *An. gambiae* the relative abundance of the two dominant populations in saliva *Proteobacteria* and *Bacteroidetes* (71.6% and 14.7% respectively, see Supplementary Table S4), and midgut (*Proteobacteria* > *Bacteroidetes* 63.5% and 36% respectively) was reversed in the salivary glands of the same mosquitoes (*Bacteroidetes* > *Proteobacteria* 54% and 26.7% respectively). The saliva of *An. gambiae* contains a higher amount of *Bacteroidetes* (14.7%), *Firmicutes* (6.9%) and *Actinobacteria* (4.6%) compared to *An. stephensi* (Figure 1A and Supplementary Table S4). We observed no discernible effect of *P. berghei* infection on the phylum level population structures in midgut, salivary glands or saliva in either *An. gambiae* or *An. stephensi* (Figure 1B and Supplementary Table S4).

At the genus level, the saliva microbiota was dominated by *Serratia* which represented greater than 50% of the bacteria in both *An. stephensi* and *An. gambiae* following a naïve blood meal (86.7% and 51.7% respectively), while the next most abundant genus *Elizabethkingia*, contributed approximately 10% in both anophelines (Figures 1C,D and Supplementary Table S5). While the bacterial composition of the saliva reflects that of the salivary glands in *An. stephensi*, with *Serratia* appearing to be the dominant bacterium (86.7% and 47.1% respectively, Figure 1C and Supplementary Table S5), this is not the case for *An. gambiae* where the relative abundance of *Serratia* was higher in the saliva (51.7%) than in the salivary glands (9.3%) (Figure 1D and Supplementary Table S5).

**Figure 2:**
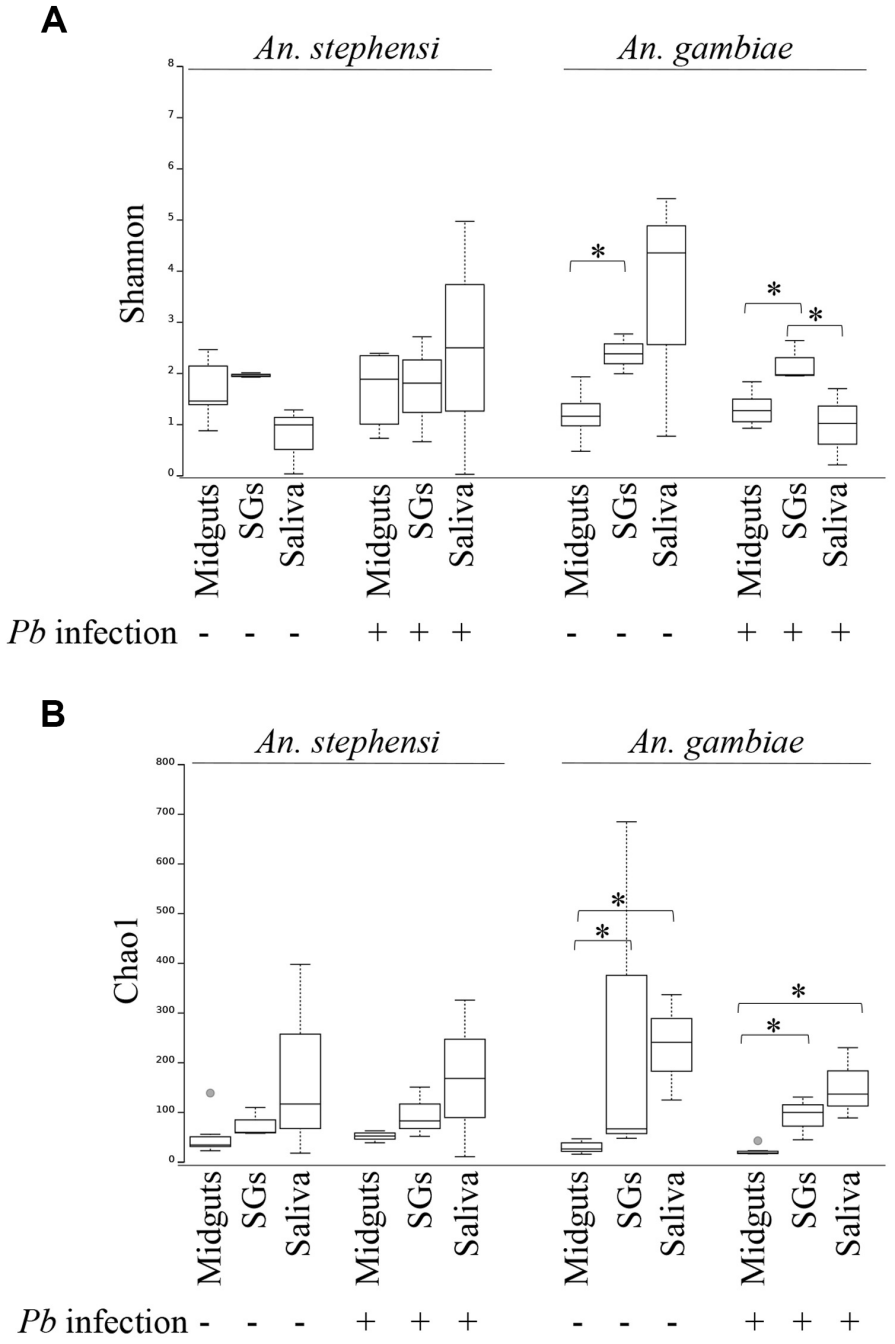
Alpha diversity of bacterial populations in different mosquito organs (midguts, salivary glands (SGs) and saliva) in the presence or absence of *P. berghei* infection. Genus level population diversity data for each sample was scored using the Shannon **(A)** or Chao1 **(B)** indexes using QIIME. Differences in those values between different organs of the same mosquito species were analysed using a Kruskal-Wallis test. Statistically significant differences (*p* < 0.05) are indicated with an asterisk.

Using two measures of population diversity, the Shannon and Chao1 indexes, we identified significant differences in the bacterial communities derived from different organs of *An. stephensi* mosquitoes while no such differences were apparent in the same organs from *An. gambiae* (Figures 2A,B). Moreover, *P. berghei* infection had no statistically meaningful impact on the overall organ-specific microbial diversity in either *Anopheline* (Figures 2A,B). We also performed a principal component analysis (PCA) to examine the relatedness of the microbial populations in different samples. In agreement with the Shannon and Chao1 alpha diversity indexes, samples from different mosquito species (Supplementary Figure S1A) and organs (Supplementary Figures S1B,C) tended to form distinct clusters reflecting their different origins, whereas organs from *P. berghei*-infected and uninfected mosquitoes tended to cluster together (Supplementary Figures S1B,C), emphasizing their similarity.

We examined our data for evidence of a specific positive association between *Serratia* abundance and *Plasmodium* infection as previously reported (Tchioffo et al., 2015). As depicted in Supplementary Figure S2, we found a trend towards increased *Serratia* abundance in the organs of *Plasmodium*-infected females. In parallel, we observed a trend towards reduced abundance of the next most prominent genus, *Elizabethkingia*. Notably, the community structure detected in a negative control sample (glucose on which no mosquitoes had fed) was qualitatively distinct at the genus level, from that containing mosquito saliva (Supplementary Figure S3), with a preponderance of low relative abundance bacterial populations which together contributed more than half the total reads. The presence of a high number of low relative abundance bacterial populations seems to be a feature of samples with low microbial biomass, as we expected our saliva-negative-control samples to be (Eisenhofer et al., 2019). *Acinetobacter, Escherichia-Shigella* and *Pseudomonas*, were identified, albeit in low abundance, in *An. gambiae* saliva (around 4% each), but this partially overlaps with the composition of the negative control, making it difficult to interpret the significance of this finding. Overall, the bacterial populations identified in the reference organs, midgut and salivary glands, of both *An. stephensi* and *An. gambiae* were in broad agreement with those previously published by ourselves (Mancini et al., 2018) and others (Sharma et al., 2014; Tchioffo et al., 2015) with *Serratia*, *Elizabethkingia*, *Acinetobacter* and *Comamonas* the most abundant genera (Figure 1 and Supplementary Table S5).

To expand the scope of our analysis beyond the 16S rRNA gene, we cultured, isolated and characterized the bacteria recovered from saliva, salivary glands and midguts of blood-fed *An. gambiae* and *An. stephensi* mosquitoes. Individual colonies were subjected to analysis using MALDI-TOF mass spectrometry, a technique which defines different bacterial populations based on genus-specific variations in the masses of peptides and small proteins, often derived from the bacterial cell surface, by comparing observed masses to genus-specific reference datasets (Raoult et al., 2015; Tandina et al., 2016). The results confirmed the presence in saliva, salivary glands and midguts of both *Anopheles* mosquitoes, of the two dominant genera, *Serratia* and *Elizabethkingia* identified by 16S rRNA gene sequencing (Table 1). At the species level MALDI-TOF analysis identified the bacteria as *Serratia marcescens and Elizabethkingia meningoseptica or Elizabethkingia miricola* that share around 98% sequence similarity with *Elizabethkingia anophelis* (not present in the instrument reference library) as previously described (Kämpfer et al., 2011).

**Table 1:** MALDI-TOF analysis of bacterial genera isolated from mosquito saliva and tissues.

Sample	*Anopheles stephensi*		*Anopheles gambiae*	
	Organism (best match)	Score Value	Organism (best match)	Score Value^c^
**Saliva**	*S. marcescens*	2.251	*S. marcescens*	2.272
			*Elizabethkingia*^b^	2.151
**SGs** ^a^	*S. marcescens*	2.334	*S. marcescens*	2.174
			*E. meningoseptica*	2.282
**Midguts**	*S. marcescens*	2.264	*S. marcescens*	2.26
	*E. meningoseptica*	2.151	*E. meningoseptica*	2.186

The table shows the bacteria isolated from An. stephensi and An. gambiae saliva, salivary glands and midguts. The bacteria were cultured and isolated using different culture media from a pool of saliva, salivary glands and midgut samples (50, 35 and 10 respectively) from blood-fed mosquitoes. The characterization was performed on at least three pure isolated colonies.

a Salivary glands.

b MALDI-TOF characterization was either Elizabethkingia meningoseptica or Elizabethkingia miricola based on MALDI Biotyper (Bruker) reference library.

c Score values > 2 are considered high confidence identifications.

### *Serratia marcescens* from mosquito saliva is transferred to a mammalian host through blood feeding

We next asked whether these bacteria might be transmissible to a mammalian host. In order to distinguish mosquito-transferred bacteria from passive transfer during biting – for example through contamination from commensal bacteria on mouse skin – we generated a transformant of *Serratia* expressing the fluorescent protein DsRed with which we could infect mosquitoes and follow its ability to colonize mosquito organs and its passage from mosquito to mouse, certain of its mosquito origin. We transformed *Serratia marcescens* isolated from salivary glands of our *An. gambiae* G3 mosquito strain. The fluorescently labelled *S. marcescens* clone, named *Serr^AgDsRed^,* was characterized by PCR and sequencing analysis, to confirm bacterial strain and modification.

**Figure 3:**
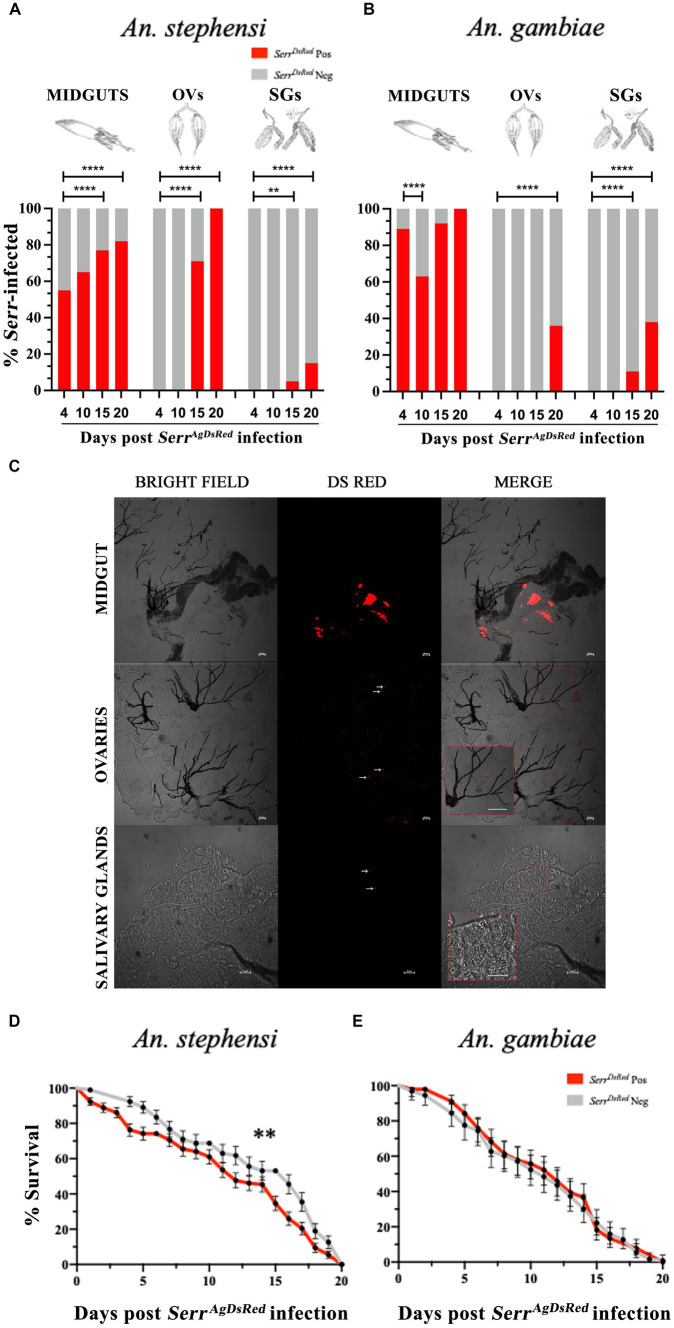
Infection of midgut, ovaries and salivary glands with fluorescent labelled *S. marcescens* varies between different organs and between anopheline species. *Serr^AgDsRed^* -fed *An. stephensi* **(A)** and *An. gambiae* **(B)** mosquitoes were dissected at day 4, 10, 15 and 20 post infection and the percentage of *Serr^AgDsRed^*- colonized *Anopheles* organs were determined by fluorescence microscopy. For each organ and time point a total of 50 mosquitoes were analyzed. The values represent the average from three independent experiments. MIDGUTs is the intestinal tract, OVs: ovaries and SGs: salivary glands. To test the relationship between the presence of DsRed bacteria in different organs and time post infection, the overall infection rate (number of DsRed positive organs vs. number of DsRed negative organs) at day 4 for each organ was compared pairwise with all other time points using a Fisher’s exact test: ^**^ < 0,01, ^****^ < 0,0001. **(C)** Representative images of *Serr^AgDsRed^* colonization of gut, ovaries (scale bar 50 μm) and salivary glands (scale bar 20 μm) in *An. gambiae* mosquitoes on a Nikon C2-Confocal microscope. Inset images show an enlargement of the region of the original, highlighted within the red square (the scale bar in the inset is the same as that in the original). **(D)** Survival curves of *An. stephensi* and **(E)** *An. gambiae* mosquitoes infected with fluorescently labelled *Serr^AgDsRed^* added to the sugar meal for 4 days. The graphs represent the mean average ± SD of three biological replicates. Each experiment was performed with 50 adult female mosquitoes. The statistical analysis was performed using a log-rank Mantel-Cox test: ^**^ *p* = 0,006.

Adult females of both anopheline species were fed on sugar solution that contained *Serr^AgDsRed^* for 4 days, and then maintained on a 6% glucose solution containing gentamicin to ensure retention of the DsRed plasmid in the transformed *Serratia.* The presence of bacteria in the gut, ovaries and salivary glands of mosquitoes fed on *Serr^AgDsRed^* -containing sugar (henceforth referred to as infection) was monitored from day 4 to day 20 post infection by fluorescence microscopy and the relationship between the number of DsRed positive organs and time post infection examined using Fisher’s exact test, taking 4 days post infection as a reference point for each organ (Figures 3A,B). Despite broad variation in the efficiency of infection in different organs, the frequency of *Serr^AgDsRed^* positivity increased over time in all tissues. Unsurprisingly, the guts of both anophelines showed the most rapid colonization and highest frequencies of DsRed positivity already surpassing 50% by day 4 post infection and reaching 100% in *An. gambiae* mosquitoes after 20 days (Figures 3A,B). In contrast, the *Serr^AgdsRed^* was slower to appear in the ovaries of infected mosquitoes, detectable starting only at day 15 (71%) in *An. stephensi* or from day 20 (36%) in *An. gambiae*, probably reflecting the time taken for bacteria to enter the hemocoel and/or penetrate tissues beyond the gut (Figures 3A,B). As observed in ovaries, *Serr^AgDsRed^* dissemination to salivary glands followed a similar time course; detectable after 15 days post infection in both *An. stephensi* (5%) and *An. gambiae* (11%) reaching 15 and 38%, respectively, by day 20 post infection (Figures 3A,B respectively). Representative images of midgut, ovaries and salivary glands from *An. gambiae* females colonized by *Serr^AgDsRed^* are shown in Figure 3C and Supplementary Figures S4, S5. Similar images of the same organs taken from negative controls fed only on sugar are presented in Supplementary Figure S6. Importantly, the presence of fluorescent-labelled *Serr^AgDsRed^* had a small but significant negative impact on *An. stephensi* survival and longevity (Figure 3D) while on *An. gambiae*, despite clear evidence of chronic infection, no negative impact was observed compared to uninfected controls (Figure 3E).

**Figure 4:**
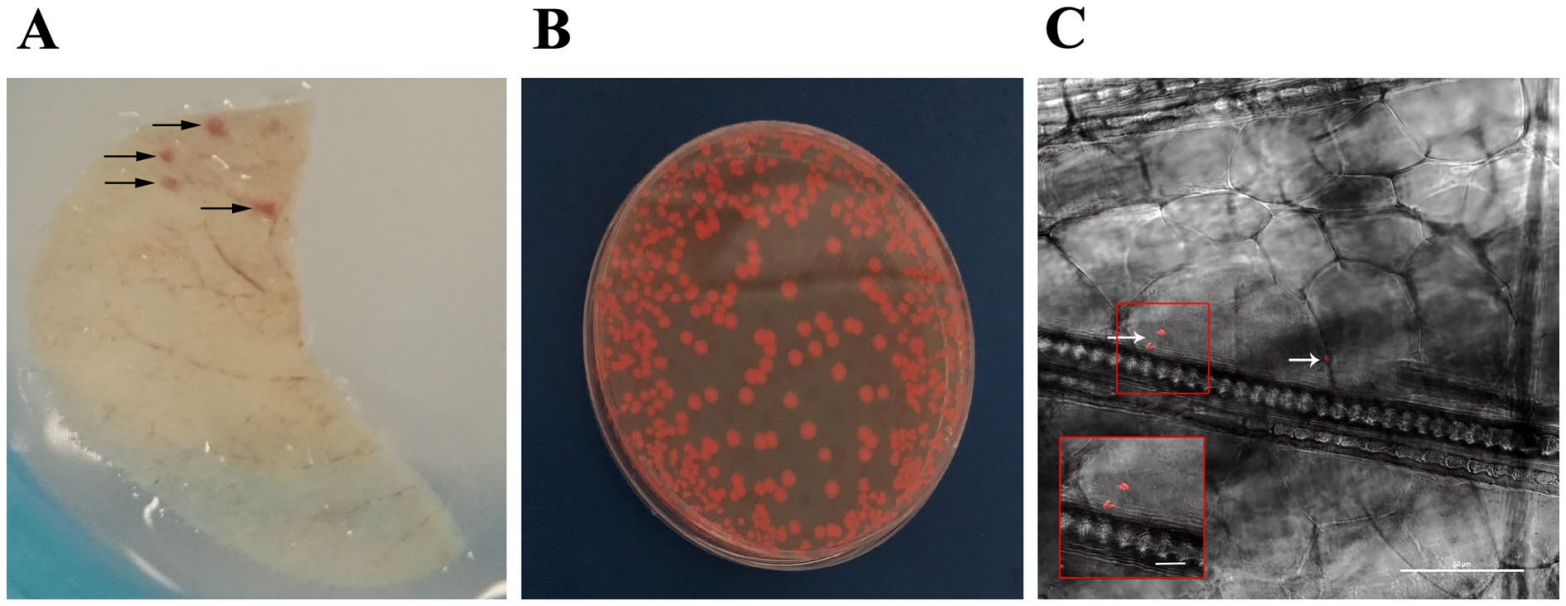
*Serr^AgDsRed^* is transmitted to mouse ear through mosquito biting. **(A)** Representative image of mouse ear after the bite of *Serr^AgDsRed^*-infected mosquitoes. Arrows indicate the areas were the feeding mosquito punctured the skin. **(B)** Representative image of *Serr^AgDsRed^* cultured in LB agar medium collected from mouse ear. **(C)** Longitudinal section of a mouse ear showing the presence of fluorescent *Serr^AgDsRed^* (white arrows) detected in the vicinity of a mosquito bite. The inset shows an enlargement of the region of the original highlighted in the red square containing the two most prominent DsRed signals. The image was collected by confocal microscopy (scale bar 50 μm).

Having demonstrated the presence of *Serr^AgDsRed^* in mosquito salivary glands (Figure 3), as well as wild-type *Serratia* in saliva itself (Figure 1 and Table 1) we went on to assess whether this mosquito-derived bacterium could be transferred to a mammalian host. Adult female *An. stephensi* and *An. gambiae* mosquitoes were fed with *Serr^AgDsRed^* as described above then maintained on sugar containing gentamicin for 18 days, by which time *Serr^AgDsRed^* could be detected in the mosquito salivary glands, and then allowed to blood feed on CD1 mouse ears (Figure 4A).

We evaluated the presence of *Serr^AgDsRed^* in mouse ears using different approaches: (i) DsRed gene amplification, (ii) bacterial culture on LB medium, and (iii) confocal microscopy. The results showed that more than half of the samples originating from ears of CD1 mice bitten by either *An. gambiae* or *An. stephensi*, analyzed by diagnostic PCR or bacterial culture, were positive for *Serr^AgDsRed^* (Table 2 and Figure 4B). Analysis using confocal fluorescence microscopy was less sensitive and, in this case, only 3 or 4 out of 9 mice were positive for *Serr^AgDsRed^* (Table 2 and Figure 4C). To examine dissemination of *Serr^AgDsRed^* to murine internal organs, liver, lung, kidney, spleen, brain and heart were recovered at 24 and 48 h post infection, homogenized and then placed in culture. Aliquots of these cultures were then examined by fluorescence microscopy. In line with previously reported data for the dissemination of *Serratia* in murine tissues (Hagihara and Sasaki, 1970), by 48 h post infection in CD1 mice, all organs except brain and spleen were positive for the presence of DsRed expressing bacteria. In similar experiments using immune compromised NOD scid gamma (NSG) mice by 24 h after infection all organs including brain and spleen showed evidence of DsRed positive bacteria (Table 3). The data indicate that the intensity of infection in lung and liver was higher than in the other organs analyzed (Table 3). As a negative control we analyzed ears and organs from mice on which mosquitoes not colonized with *Serr^AgDsRed^* were allowed to feed. All mice tested negative for the presence of *Serr^AgDsRed^*.

**Table 2:** *Serratia^*AgDsRed*^* is transferred from mosquitoes to mice ears during blood feeding.

Mosquito species	Infected mouse/ Total mice		
	PCR^a^	Culture^b^	Microscopy^c^
*An. stephensi*	4/9	4/9	3/9
*An. gambiae*	6/9	6/9	4/9

*Serratia^AgDsRed^*-infected mosquitoes were allowed to blood feed on CD1 mice. Mouse ears were and processed immediately after mosquito blood feeding.

a Diagnostic PCR for Ds-Red was performed on DNA extracted from ears of infected mice.

b The ears of infected mice were homogenized, and bacteria grown in culture medium;

c The ears of infected mice were longitudinally dissected and analyzed by confocal microscopy for the presence of DsRed fluorescent Serratia. A total of 9 mice was used for both mosquito species in three independent experiments.

**Table 3:** *Serratia^*AgDsRed*^* colonization of mice organs.

Organs	Time post *Serratia^AgDsRed^* infection in NSG mice				
	24 h			48 h	
	CFU^a^	Culture^b^		CFU	Culture
Lung	5.5×10^3^	+		8.0×10^3^	+
Liver	7.9×10^3^	+		6.0×10^3^	+
Kidney	2.9×10^2^	+		3.0×10^2^	+
Spleen	9.2×10^2^	+		5.4×10^2^	+
Brain	ND	+		9.0×10^2^	+
Heart	ND	+		6.0×10^2^	+

a Colony forming units (CFU) were counted. A total of 4 mice were used in two independent experiments.

b Culture medium was analyzed by fluorescence microscopy and described as positive (+) if DsRed expressing bacteria were detected. ND = Not Done.

### *Serratia* is transmitted to the mammalian host together with *Plasmodium berghei* during blood feeding

Having shown that *Serratia* is the most abundant bacteria present in the salivary gland and saliva of the *P. berghei*-infected mosquitoes (Figsures 1C,D) as well as mosquito-to-mouse transfer of *Serratia* through the saliva we wanted to know if the microbe is released together with the parasite during blood feeding and whether the organ targeted by the parasite might also host the bacterium following an infected bite. Therefore, *An. gambiae* mosquitoes were co-infected with *Serr^AgDsRed^* and a *P. berghei* line expressing green fluorescent protein (GFP) named Bergreen (Matz et al., 2013) and 18 days post *P. berghei* infection, were allowed to blood feed on CD1 mice. Forty-eight hours post blood feeding we performed a diagnostic PCR on DNA extracted from the liver of animals on which the mosquitoes had fed, looking for the presence of both GFP and DsRed genes. Both GFP and DsRed were detected in the livers of all six of the infected animals. These results show that *Serratia* and *P. berghei* were released together during natural blood feeding and were able to infect the liver of 100% of the mice analyzed (Supplementary Figure S7).

## Discussion

The saliva of anophelin*e* mosquitoes represents the medium in which *Plasmodium* parasites are transmitted to a new host while the protein constituents of saliva are themselves targeted by the host immune response as well as shaping the response to which invading sporozoites are exposed (Donovan et al., 2007). Indeed, mosquito saliva has been exploited to develop innovative strategies to block malaria transmission (Yamamoto et al., 2010) (Yamamoto et al., 2012; Sumitani et al., 2013). Thus, our finding that mosquito saliva contains and transmits bacteria to a mammalian host not only extends the influence of the mosquito microbiome to encompass interactions between parasite and mammalian host but also opens the possibility of using paratransgenic commensal mosquito bacteria as a vaccine delivery strategy.

Using 16S rRNA gene sequencing we identify a core salivary microbiome, common to both *An. stephensi* and *An. gambiae* that is dominated by *Serratia* with a smaller contribution from *Elizabethkingia*. This finding is also supported by an independent analysis using MALDI-TOF, a complementary, culture-dependent method of bacterial identification. It is noteworthy that this population structure mirrors that detected in the salivary glands of this study, consistent with the possibility that the bacterial populations detected in the saliva originate in the salivary glands. Moreover, the community structure we identify in salivary glands matches well with several similar published analyses of the same tissue in a range of anopheline and non-anopheline mosquitoes (Gimonneau et al., 2014; Tchioffo et al., 2015; Mancini et al., 2018).

Having established the predominance of wild type *Serratia* in both salivary glands and saliva itself we show that a transformant of the same bacterium expressing the fluorescent marker, DsRed, could be introduced *via* the sugar solution on which the mosquitoes fed and was subsequently detected in their salivary glands. It was notable that the colonization of the salivary glands by *Serratia^AgDsRed^* took longer and reached a lower frequency than that observed in the midgut. Moreover, we cannot rule out the possibility that the dissemination of *Serratia^AgDsRed^* was favored by the presence in the sugar solution of gentamicin, reducing competition from other bacteria. Thus while the fact of colonization of the salivary glands by wild type *Serratia* is amply supported by both 16S rRNA sequencing and MALDI-ToF (Figure 1 and Table 1) the rate and extent of its dissemination beyond the midgut may be imperfectly reflected by *Serratia^AgDsRed^.* Furthermore, while all mosquitoes were exposed to the same initial concentrations of *Serratia^AgDsRed^*, the extent of their ingestion will have varied between individuals. Thus, despite the fact that the frequency of *Serratia^AgDsRed^* infection of midguts was high, the intensity of that infection may have varied significantly. This could explain the reduced frequency of *Serratia^AgDsRed^* in salivary glands (i.e., only the highest intensity midgut infections disseminated to the salivary glands). In future studies it will be interesting to examine the impact of ingesting differing starting concentrations *Serratia^AgDsRed^* on the rate and extent of its conlonization of distal organs.

Importantly, and consistent with previous studies (Ezemuoka et al., 2020), infection with fluorescent *Serratia* had little (*An. stephensi*) or no (*An. gambiae*) negative impact on mosquito survival. In addition, transfer of fluorescent *Serratia* to the mammalian host was observed by confocal microscopy. The DsRed marker gene was also detected by PCR while viable fluorescent bacteria were cultured from extracts of both internal organs (liver, lung, kidney, spleen, brain and heart) and ears of mice on which the infected mosquitoes had fed. While we cannot formally exclude mechanical transfer of bacteria, the transgenic nature of this bacterium, its presence in salivary glands and the dependence of its detection in murine tissues on blood feeding with infected mosquitoes, leaves transfer through saliva during blood feeding as the most credible route of transmission.

Thus, together, our findings demonstrate the potential of *An. gambiae* to act as a biological bacterial vector through injected saliva consistent with previous reports of *Rickettsia felis* in *An. gambiae* (Dieme et al., 2015) and in common with several other blood feeding arthropods such as ticks, lice and fleas (Victoria and Yermia Sem, 2021). Indeed, given the established role of *Serratia* in multidrug-resistant bacterial infections (Iguchi et al., 2014) and the mosquito to mammalian transfer of *Serratia* shown here, the possible role of mosquitoes as a source of such infections seems worthy of further investigation.

The predominance of *Serratia* identified in the salivary microbiome may also be relevant to plasmodium transmission blocking efforts. *Serratia* belongs to a family of bacteria originally identified within the ovaries and has been shown to exhibit vertical transmission (4). Moreover, both naturally occurring (Gao et al., 2021) and engineered transgenic (Wang et al., 2012) strains of the same bacterial family have been successfully deployed to block malaria transmission. *Serratia* therefore represents a strong candidate for transmission blocking strategies that can be passed from one generation to the next. Our data extend the potential influence of the bacterium to encompass immune responses to *Plasmodium* in a mammalian host, akin to that envisaged in the ‘flying vaccine’ concept (Yamamoto et al., 2010).

Our data also support a possible interaction between the *Serratia* and *Elizabethkingia* population in different organs, and the *Plasmodium* parasite. We find a trend towards increased abundance of *Serratia* but reduced abundance of *Elizabethkingia* in almost all tissues analyzed (midgut, salivary glands and saliva) following a blood meal infected with *P. berghei*. Notably, our microbiome analyses of mosquito organs were performed at time points at which *Plasmodium* oocysts (in the midgut) and sporozoites (in the salivary glands) were most abundant. While the high abundance of *Elizabethkingia* in our samples may be an artifact of their laboratory origin (Boissière et al., 2012), in the case of *Serratia* our findings are in good agreement with field studies (Tchioffo et al., 2015). Field-derived *An. gambiae* mosquitoes infected with *P. falciparum* also exhibited an infection-induced increase in *Serratia* abundance in both midgut and salivary glands (Tchioffo et al., 2015). Interestingly, these studies measured the microbiome of mosquito organs at day 7 post infection, a time point at which while oocysts will be well developed in the midgut, sporozoites may not be present in the salivary glands. These data are therefore consistent with the possibility that changes in the bacterial communities in the salivary glands may be secondary to changes observed in the midgut, emphasizing the well documented communication between bacterial populations in different organs (Balaji et al., 2021).

Given the many and complex ways in which mosquitoes, parasites and the mosquito microbiome can interact (Vinayagam et al., 2023) the precise mechanisms underpinning the apparent positive association between *Plasmodium* and *Serratia* await further study. However, the finding of the same trend in both field and lab-raised *An. gambiae,* infected with different species of *Plasmodium,* strengthens the possibility that this interaction is real and that it may confer some fitness advantage to the mosquito (Bando et al., 2013; Chen et al., 2017; Bai et al., 2019). Counter-intuitively, the presence of *Serratia* has also been linked to a significant inhibition of *Plasmodium* infection through blockade of ookinete invasion of the midgut epithelium (Bando et al., 2013; Tchioffo et al., 2013). However, this effect was dependent on the particular strain of *Serratia* used; notably, a specifically mosquito-adapted *Serratia* strain lost this *Plasmodium* inhibitory capacity (Bando et al., 2013). To resolve this apparent paradox, it will be interesting to examine the possible existence of multiple strains of *Serratia* in anopheline mosquitoes and characterize their distribution in different organs, as has been performed previously for *Asaia* (Chouaia et al., 2010). These questions assume particular importance when considering lab-raised and field-caught anopheline mosquitoes. While both contain *Serratia* (Tchioffo et al., 2015; Mancini et al., 2018), differences in the strain of *Serratia* present in these two environments are likely. Moreover, it is well established that some components of the mosquito microbiome such as Elizabethkingia referred to above, are abundant in lab-reared mosquitoes but almost absent in field samples (Boissière et al., 2012). Thus, it will be important in future studies to examine the dissemination and possible transmission of field-derived *Serratia* strains in the context of a microbiome reconstituted to more accurately reflect field conditions.

In sum, our data demonstrate the presence of bacteria in the saliva of *An. gambiae* and *An. stephensi*, two of the most important vectors for malaria transmission in Africa and Asia, respectively. The fact that these bacteria are transferred through the saliva during blood feeding has implications for our understanding of mosquitoes as putative vectors of bacterial infection while also highlighting novel approaches to counter *Plasmodium* transmission or boost resistance to medically important, zoonotic infectious agents in mammalian reservoir populations.

## Data availability statement

The datasets presented in this study can be found in online repositories. The names of the repository/repositories and accession number(s) can be found in the article/Supplementary material.

## Ethics statement

The animal study was reviewed and approved by Italian Ministry of Health.

## Author contributions

RS, GF, and AA designed the experiment. AA, CD, AC, GI, GM, AT, and FD’A carried out the experiments and collected the data. AA, CD, and RS analysed the data. EN performed metagenomic analysis. AA, MP, and RS wrote the paper. All authors contributed to the article and approved the submitted version.

## Funding

This work was supported by the Italian Ministry of University and Research (MUR) by Programmi di Ricerca Scientifica di Rilevante Interesse Nazionale (PRIN), grant no. 2015JXC3JF_003.

## Conflict of interest

The authors declare that the research was conducted in the absence of any commercial or financial relationships that could be construed as a potential conflict of interest.

## Publisher’s note

All claims expressed in this article are solely those of the authors and do not necessarily represent those of their affiliated organizations, or those of the publisher, the editors and the reviewers. Any product that may be evaluated in this article, or claim that may be made by its manufacturer, is not guaranteed or endorsed by the publisher.

## Footnotes

^1^ https://www.beiresources.org/Portals/2/VectorResources/2016%20Methods%20in%20Anopheles%20Research%20full%20manual.pdf

^2^ https://www.pberghei.eu
