# Supplementary material for: Anopheline mosquito saliva contains bacteria that are transferred to a mammalian host through blood feeding
Source: Front Microbiol. 2023 Jul 18;14:1157613. doi: 10.3389/fmicb.2023.1157613 (PMC10392944; doi:10.3389/fmicb.2023.1157613)
Supplement: Supplementary file 1 [file Data_Sheet_1.zip › Accoti et al. Supplementary material.pdf]

Supplementary material

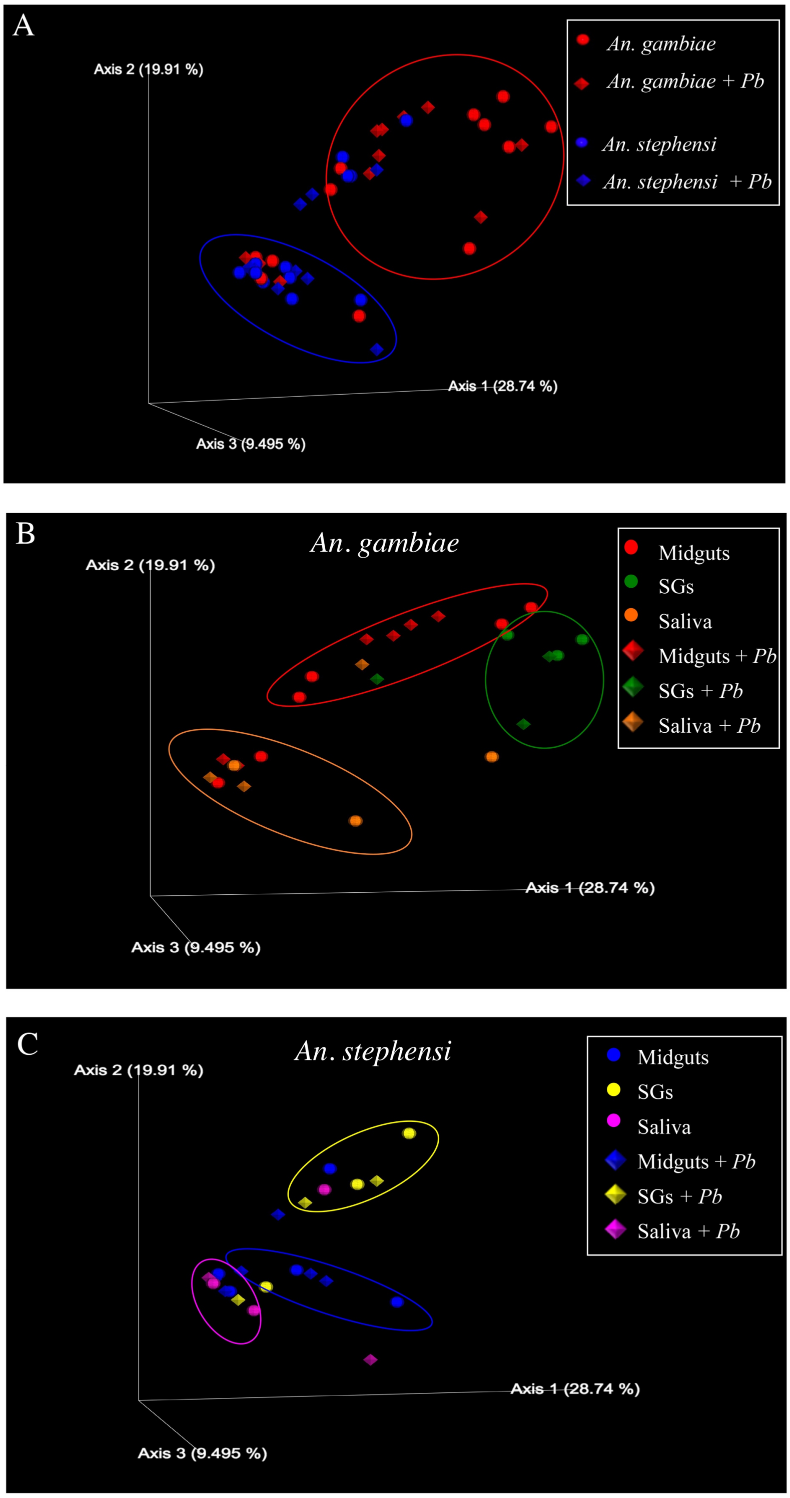

Supplementary Figure S1: Tridimensional principal coordinate analysis (PCoA) profile of microbial diversity across all *An. gambiae* and *An. stephensi* tissues using Bray Curtis metric. Total organs from *P.berghei* (*Pb*)-infected and non-infected *An. gambiae* and *An. stephensi* mosquitoes (A); *An. gambiae* and *An. stephensi* midguts, salivary glands (SGs) and saliva from non-infected and *P. berghei*-infected mosquitoes (B and C respectively). Samples belong to three independent replicates.

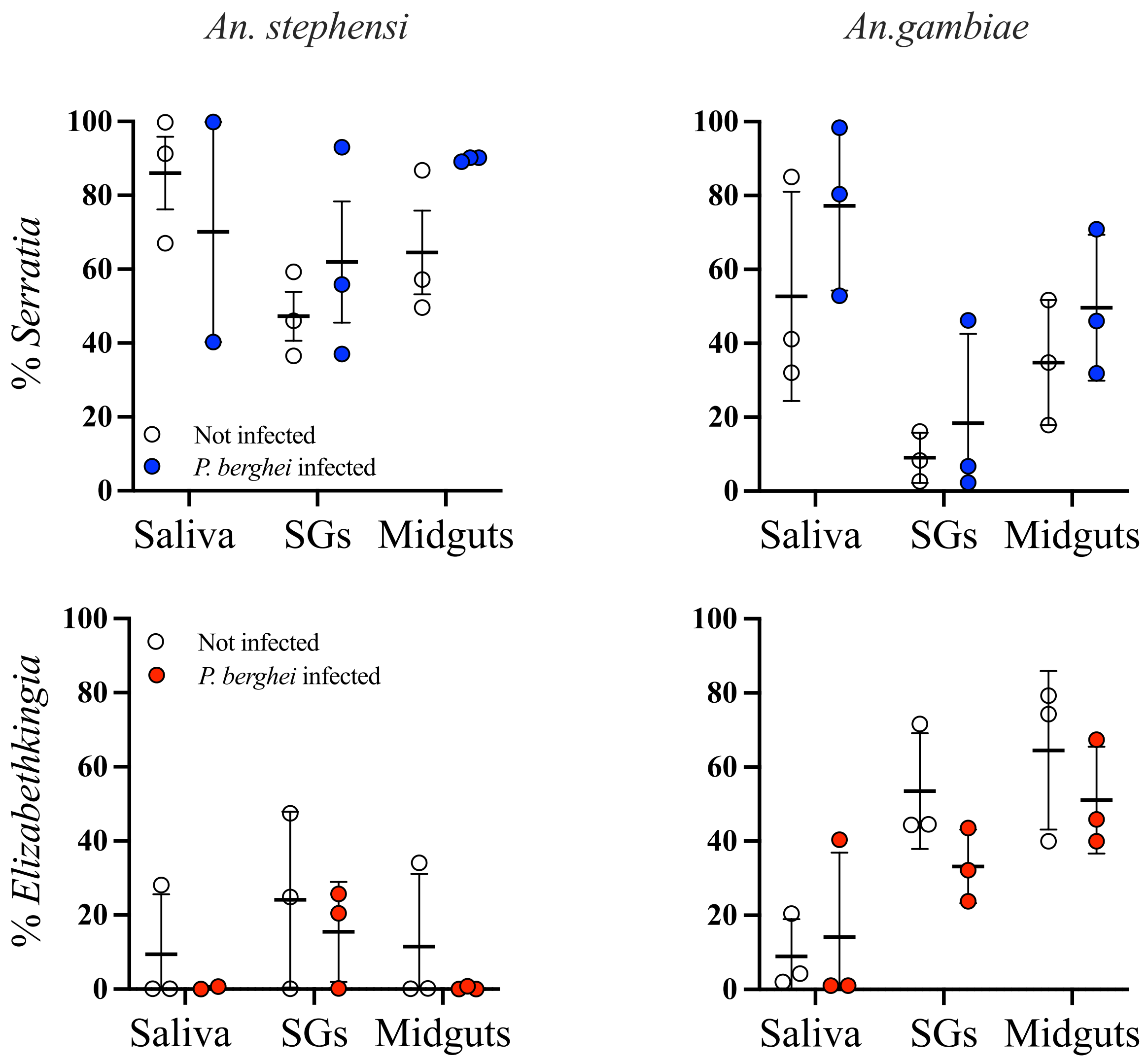

Supplementary Figure S2. *P. berghei* infection influences *Serratia* and *Elizabethkingia* load in *An. gambiae* and *An. stephensi* midguts, salivary glands and saliva. The graphs show the relative abundance (% of total bacteria) of *Serratia* and *Elizabethkingia* in the saliva, salivary glands (SGs) and midguts, of *An. stephensi* and *An. gambiae* mosquitoes infected, or not, with *P. berghei,* analyzed by 16S rRNA sequencing. The values represent the mean ± SD of three independent replicates. Differences between unifected and *P.berghei*-infected samples were found to be not significant.

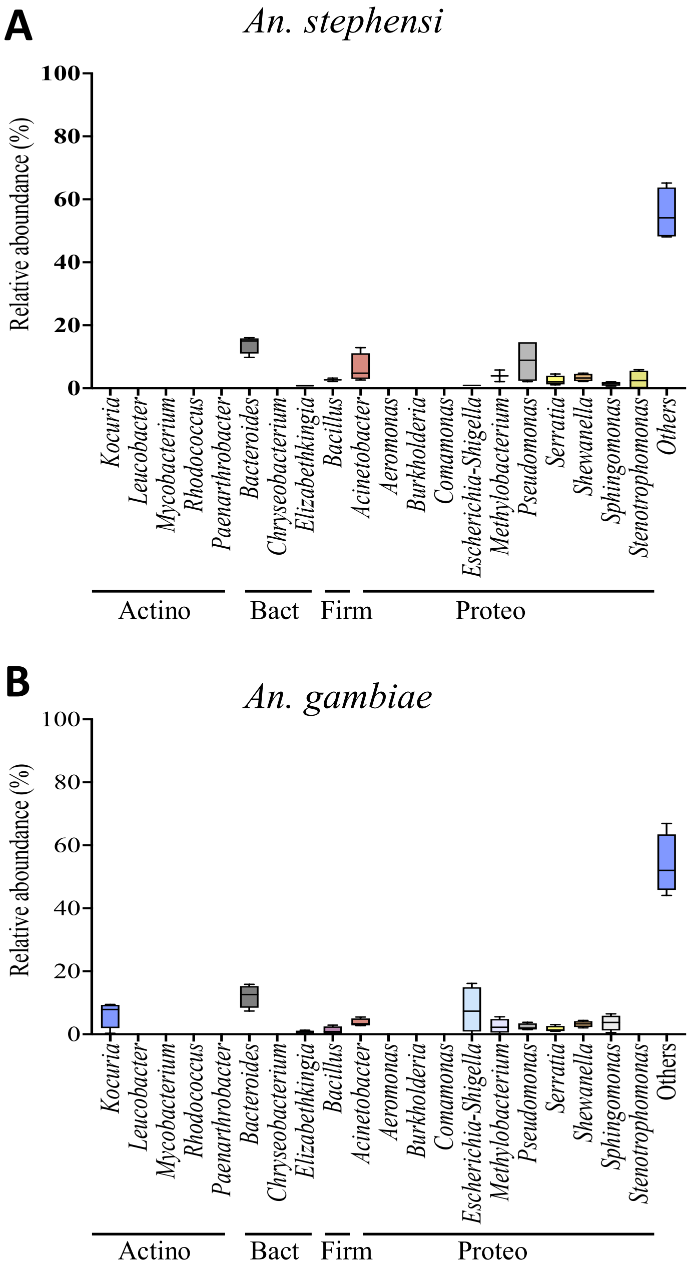

Supplementary Figure S3. Relative abundance (% total) of bacteria present in the glucose solution used to collect mosquito saliva. Taxonomic classification at the phylum and genus level of bacterial relative abundance in the glucose solution used to collect mosquito saliva (negative control sample). All the bacteria that were not identified and/or had a relative abundance lower than 0.5% were grouped together as “Other”. Actino: *Actinobacteria*, Bact: *Bacteroidetes*, Firm: *Firmicutes* and Proteo: *Proteobacteria*. The values shown represent the median ± SD of three independent experiments.

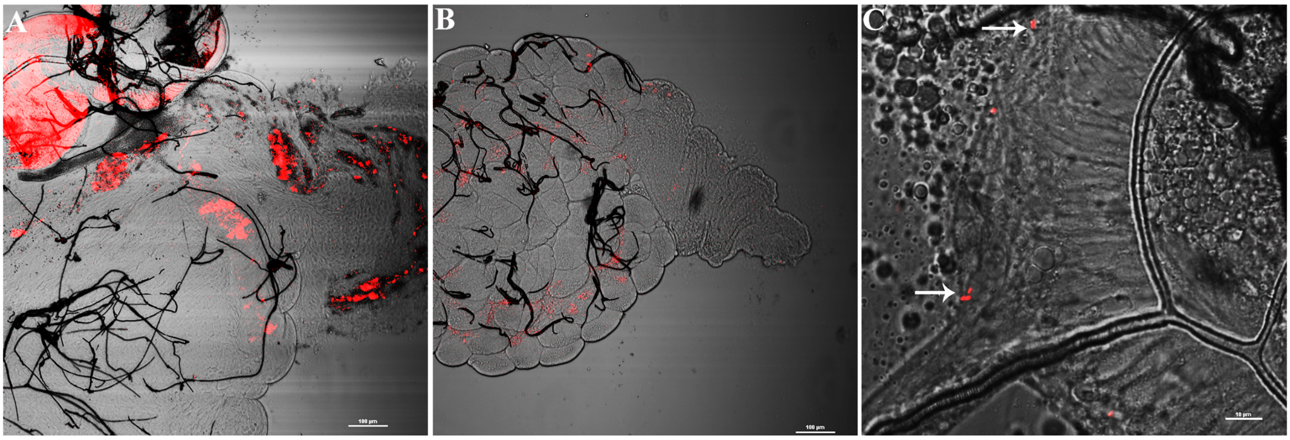

Supplementary Figure S4. Representative images of dsRed-expressing *Serratia* in mosquito tissues. Representative merged bright field/DsRed images of gut (A, scale bar 100 μm), ovaries (B, scale bar 100 μm), and salivary glands (C, scale bar 10 μm); in *An. gambiae* mosquitoes fed with sugar solution containing and $Serr^{AgDsRed}$. Arrows indicate $Serratia^{AgDsRed}$ bacteria. All images taken with a Nikon C2- 528 Confocal microscope.

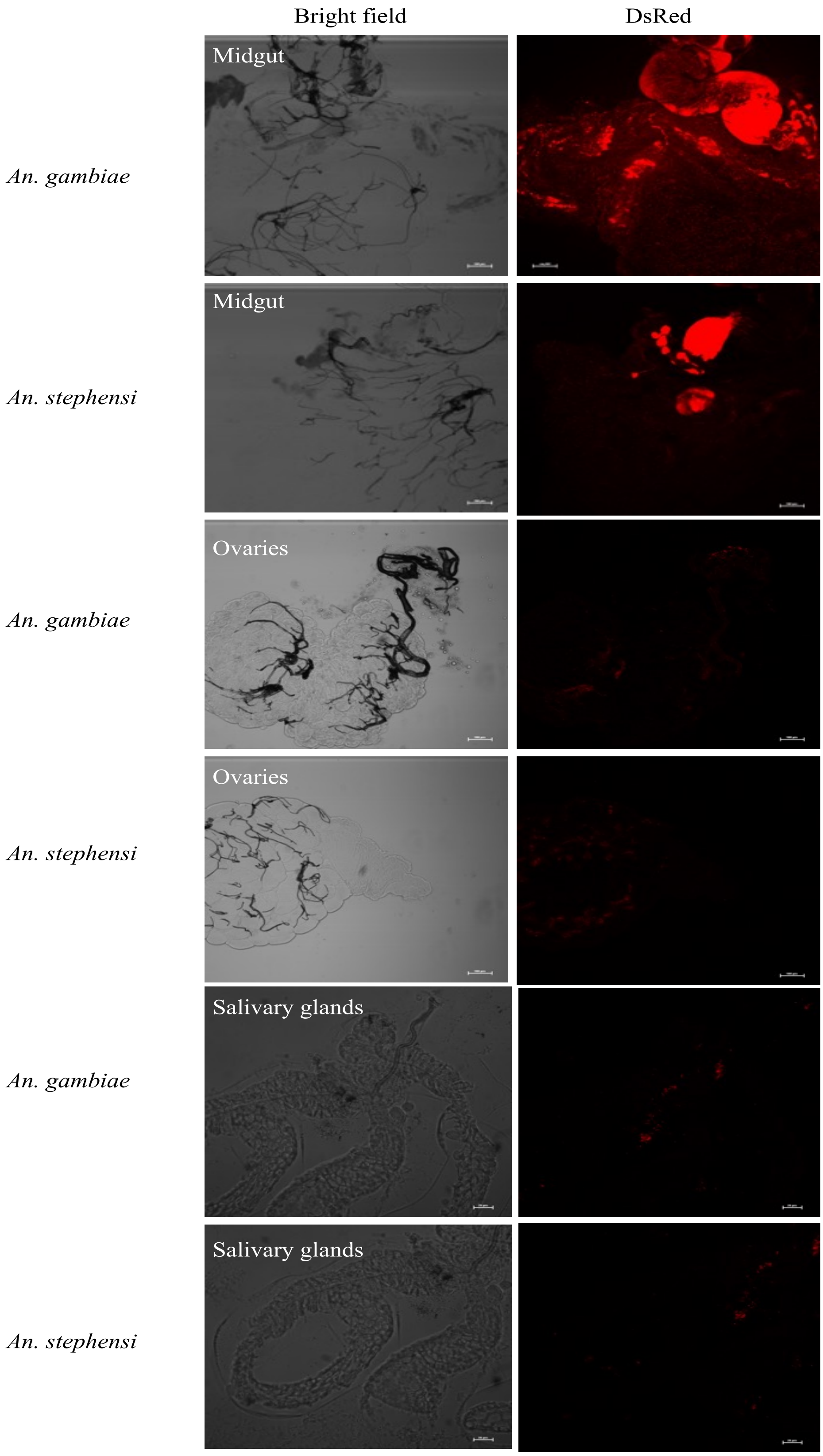

Supplementary Figure S5. Additional representative images of dsRed-expressing *Serratia* in mosquito tissues. Representative bright field or DsRed fluorescence images of midguts (scale bar 100 μm), ovaries (scale bar 100 μm), and salivary glands (scale bar 10 μm) from *An. gambiae* and *An. stephensi* mosquitoes fed with sugar solution containing and $Serr^{AgDsRed}$. All images taken with a Nikon C2- 528 Confocal microscope.

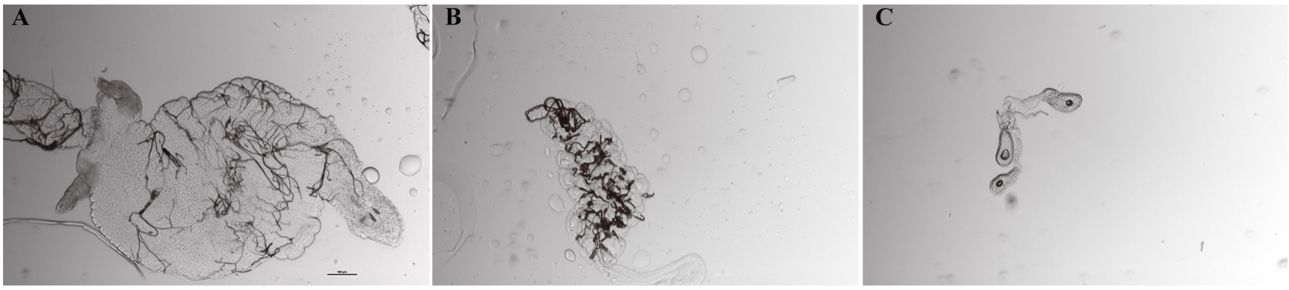

Supplementary Figure S6. Lack of DsRed-expressing *Serratia* in not infected sugar-fed tissues. Representative merged bright field/DsRed images of gut (A, scale bar 100 µm), ovaries (B), and salivary glands (C) in *An. gambiae* mosquitoes fed with only sugar solution. All images taken with 10x magnification on a Nikon C2- 528 Confocal microscope.

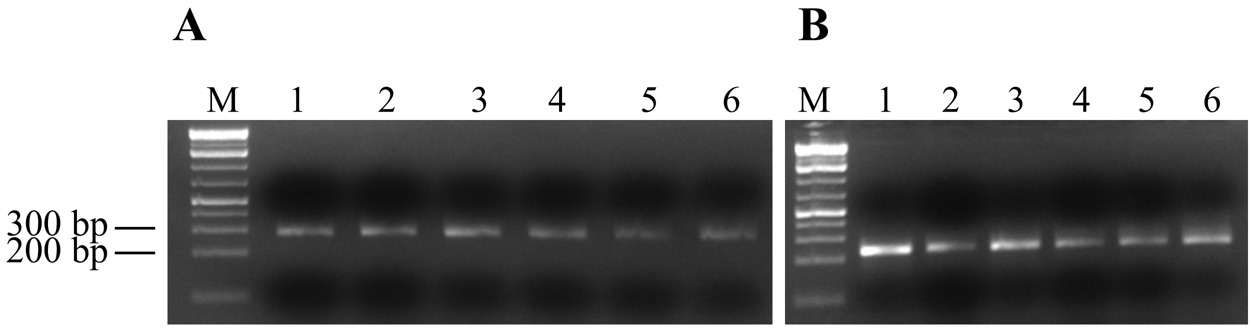

Supplementary Figure S7. Diagnostic PCR from mouse liver co-infected with *P. berghei* and *Serr*^{AgDsRed}. Representative images of PCR amplification signal from DNA extracted from the liver of six different mice (lane 1-6) co-infected with *P. berghei* Bergreen parasites and *Serr*^{AgDsRed}. (A) The expected band of 300 bp was obtained using primers specific for the *green fluorescent protein* (*GFP*) gene expressed by the transgenic *P. berghei*. (B) In the same samples, the expected band of ~270 bp was generated using primers specific for the *DsRed* gene expressed by the transgenic *Serratia*^{AgDsRed}. M: 1kb plus marker.

Table S1. Summary of samples analyzed for metagenomic analysis

Mosquito species	Sample type	*P. berghei* infection (Y/N)[a]	N°. of replicates[b]
An. gambiae	Midgut[c]	N	3
An. gambiae	SGs[d]	N	3
An. gambiae	Saliva[e]	N	3
An. gambiae	Saliva negative control[f]	N	3
An. stephensi	Midgut	N	3
An. stephensi	SGs	N	3
An. stephensi	Saliva	N	3
An. stephensi	Saliva negative control	N	2
An. gambiae	Midgut	Y	3
An. gambiae	SGs	Y	3
An. gambiae	Saliva	Y	3
An. gambiae	Saliva negative control	Y	2
An. stephensi	Midgut	Y	3
An. stephensi	SGs	Y	3
An. stephensi	Saliva	Y	2
An. stephensi	Saliva negative control	Y	2

[a]: Y/N signifies samples that are infected (Y) or not infected (N) with *Plasmodium berghei* parasites; [b]: N°. of biological replicates; [c]: 10 midguts were collected at day 14 post-blood feeding for each replicate; [d]: 35 salivary glands were collected at day 21 post-blood feeding for each replicate; [e]: Saliva was collected from 50 mosquitoes at day 21 post-blood feeding for each replicate; [f]: saliva negative control represents a mosquito-free aliquot of the sugar solution used to feed the mosquitoes.

Table S2. Sequencing outputs and proportion of reads used for downstream analysis

Sample	Sample-id	Input	Filtered	Denoised	Merged	Non-chimeric
An. stephensi BF* - gut	100_S1_L001	569710	522267	522267	494548	358316
An. stephensi BF - salivary glands	102_S3_L001	435871	399883	399883	383180	333979
An. stephensi BF - saliva	103_S4_L001	614893	567874	567874	556752	544076
An. stephensi IBF**- gut	105_S5_L001	668232	618471	618471	592174	406890
An. stephensi IBF - salivary glands	107_S7_L001	554203	508808	508808	496736	477791
An. gambiae BF - gut	109_S8_L001	509593	476118	476118	468375	423279
An. gambiae BF - salivary glands	111_S10_L001	605649	562896	562896	548361	455096
An. gambiae BF - saliva	112_S11_L001	556191	512491	512491	495681	487431
An. gambiae BF - saliva negative control	113_S12_L001	552787	505327	505327	482603	463471
An. gambiae IBF - gut	114_S13_L001	524911	482728	482728	473021	392394
An. gambiae IBF - salivary glands	116_S15_L001	423308	385922	385922	369282	290276
An. gambiae IBF - saliva	117_S16_L001	498997	459813	459813	449775	432794
An. stephensi BF - gut	144_S1_L001	322810	314215	314215	306059	216865
An. stephensi BF - salivary glands	146_S3_L001	387083	378590	378590	374953	306866
An. stephensi BF - saliva	147_S4_L001	358927	349913	349913	349015	348663
An. stephensi BF - saliva negative control	148_S5_L001	428926	419071	419071	403665	363148
An. stephensi IBF - gut	149_S6_L001	299346	291392	291392	283510	210468
An. stephensi IBF - salivary glands	151_S8_L001	335442	328161	328161	325576	269857
An. stephensi IBF - saliva	152_S9_L001	306940	298368	298368	297633	297537
An. stephensi IBF - saliva negative control	153_S10_L001	344503	336070	336070	323688	306101
An. gambiae BF - gut	154_S11_L001	413854	406119	406119	404637	386627
An. gambiae BF - salivary glands	156_S13_L001	281162	274418	274418	265599	255382
An. gambiae BF - saliva	157_S14_L001	319310	310014	310014	302279	274493
An. gambiae BF - saliva negative control	158_S15_L001	290524	279216	279216	267648	239049
An. gambiae IBF - gut	159_S16_L001	293689	286984	286984	284992	227494
An. gambiae IBF - salivary glands	161_S18_L001	236967	230849	230849	227092	198408
An. gambiae IBF - saliva	162_S19_L001	343776	335300	335300	333127	329095
An. gambiae IBF - saliva negative control	163_S20_L001	395492	385725	385725	375622	336974
An. gambiae IBF - saliva negative control	164_S21_L001	423834	411725	411725	397789	369046
An. stephensi BF - gut	169_S1_L001	517753	489077	489077	483555	475684
An. stephensi BF - salivary glands	171_S3_L001	436756	419845	419845	410968	368076
An. stephensi BF - saliva	172_S4_L001	514664	496329	496329	490270	467719
An. stephensi BF saliva negative control	173_S5_L001	692945	668704	668704	643559	601297
An. stephensi IBF - gut	174_S6_L001	476992	459974	459974	454937	406316
An. stephensi IBF - salivary glands	176_S8_L001	424163	409352	409352	399340	328559
An. stephensi IBF - saliva	177_S9_L001	592158	559396	559396	539194	511343
An. stephensi IBF - saliva negative control	178_S10_L001	471433	453747	453747	438176	420881
An. gambiae BF - gut	179_S11_L001	524093	505548	505548	496529	464630
An. gambiae BF - salivary glands	181_S13_L001	371609	354701	354701	346795	311093
An. gambiae BF - saliva	182_S14_L001	298341	282494	282494	277304	270037
An. gambiae BF - saliva negative control	183_S15_L001	354589	331723	331723	305746	290126
An. gambiae IBF - gut	184_S16_L001	404213	388952	388952	384022	368201

An. gambiae IBF - salivary glands	186_S18_L001	266491	255960	255960	250900	232285
An. gambiae IBF - saliva	187_S19_L001	473083	451972	451972	444441	409019

BF*: blood feeding

IBF**: infected blood feeding with *P.berghei*

Table S4. Relative abundance (% total) of bacteria at the phylum level in *Anopheline* saliva and tissues

Mosquito strain	*P. berghei* infection	Tissue	*Actinobacteria*	*Bacteroidetes*	*Firmicutes*	*Proteobacteria*	Other
An. stephensi	N	Saliva	0.5 ± 0.6	9.7 ± 16.1	1.2 ± 1	88.4 ± 16.4	0.4 ± 0.6
	N	SGs[a]	4.8 ± 4.4	27.5 ± 28.8	3.8 ± 6.4	63.8 ± 23.6	0.0
	N	Midgut	4.1 ± 3.6	15.4 ± 17.3	0.0	80.4 ± 18.8	0.0
An. stephensi	Y	Saliva	3.1 ± 5.2	3.9 ± 5.7	6.4 ± 10.5	85.6 ± 22.8	0.9 ± 1.3
	Y	SGs[a]	9.0 ± 8.7	20.1 ± 19.1	0.2 ± 0.2	70.5 ± 24.0	0.0
	Y	Midgut	2.8 ± 3.2	2.9 ± 4.2	0.1 ± 0.2	93.9 ± 3.3	0.0
An. gambiae	N	Saliva	4.6 ± 3.1	14.7 ± 14.1	6.9 ± 3.9	71.6 ± 21.6	2.3 ± 2.4
	N	SGs[a]	13.3 ± 19	54.0 ± 16.4	5.5 ± 4.8	26.7 ± 16	0.4 ± 0.6
	N	Midgut	0.4 ± 0.6	36.0 ± 40.1	0.0	63.5 ± 40.7	0.0
An. gambiae	Y	Saliva	0.2 ± 0.1	14.2 ± 24.1	0.6 ± 0.6	84.5 ± 25.0	0.3 ± 0.3
	Y	SGs[a]	21.3 ± 20.3	34.0 ± 11.2	6.3 ± 10.0	37.9 ± 25.1	0.3 ± 0.2
	Y	Midgut	0.2 ± 0.2	42.1 ± 36.7	0.0	57.6 ± 36.8	0.0

[a] SGs: salivary glands. The values shown represent the mean ± SD of three independent experiments. All the bacteria that were not identified and/or resulted in a relative abundance lower than 0.4% were labelled “Other”.

Table S5. Relative abundance (% total) of bacteria at the genus level in *Anopheline* saliva and tissues

Bacteria	Tissue	*An. stephensi* BF[a]	*An. stephensi* *Pb* infected[b]	*An. gambiae* BF	*An. gambiae* *Pb* infected
	**Saliva**				
Acinetobacter	"	0.4 ± 0.8	1.3 ± 1.6	4 ± 5	0.7 ± 0.6
Bacillus	"	0.3 ± 0.4	0.8 ± 1.1	ND[c]	0.1 ± 0.2
Elizabethkingia	"	9.5 ± 16.3	0.4 ± 0.5	9.8 ± 11.4	13.6 ± 23.2
Escherichia-Shigella	"	0.3 ± 0.5	0.1 ± 0.1	0.1 ± 0.2	0.1 ± 0.2
Leucobacter	"	ND	2.0 ± 2.8	0.4 ± 0.4	0.0 ± 0.0
Pseudomonas	"	ND	5.5 ± 7.7	4.5 ± 6.3	0.1 ± 0.1
Serratia	"	86.7 ± 16.4	65.1 ± 49.2	51.7 ±	77.2 ± 22.9
Stenotrophomonas	"	ND	2.2 ± 3.0	ND	5.3 ± 9.2
Others		2.6 ± 2.5	22.6 ± 5.6	25 ± 20	2.7 ± 0.6
	**Salivary glands**				
Acinetobacter	"	12.9 ± 21.9	7.4 ± 12.0	2.6 ± 3.3	4.1 ± 5.3
Bacillus	"	4 ± 6.9	0.2 ± 0.3	3.2 ± 5.4	6.0 ± 10.3
Comamonas	"	0 ± 0.1	0.0 ± 0.1	2.4 ± 4	0.8 ± 1.3
Elizabethkingia	"	26 ± 26.5	15.5 ± 13.5	54.8 ±	33.2 ± 9.9
Escherichia-Shigella	"	6.2 ± 10.7	0.3 ± 0.5	8.3 ± 14.2	11.8 ± 20.3
Leucobacter	"	0.5 ± 0.8	0.8 ± 1.4	1.4 ± 2.3	0.1 ± 0.2
Paenarthrobacter	"	1.8 ± 3.1	4.4 ± 7.7	10.6 ±	13.9 ± 24.0
Pseudomonas	"	0.3 ± 0.5	0.2 ± 7.7	0.2 ± 0.2	0.0 ± 0.1
Serratia	"	47.1 ± 15.3	62.0 ± 28.5	9.3 ± 7.2	18.4 ± 24.2
Stenotrophomonas	"	0.5 ± 0.8	0.0 ± 0.1	1.3 ± 2.1	1.4 ± 2.5
Others		0.7 ± 0.2	9.3 ± 2.6	5.9 ± 3.1	10.3 ± 2.6
	**Midguts**				
Acinetobacter	"	2.3 ± 2.4	4.7 ± 6.3	6.2 ± 10.7	0.4 ± 0.4
Comamonas	"	3.2 ± 5.5	6.8 ± 11.8	8.2 ± 14.2	3.3 ± 3.6
Elizabethkingia	"	11.5 ± 19.7	0.3 ± 0.5	36.1 ±	42.1 ± 36.7
Paenarthrobacter	"	ND	0.1 ± 0.1	0.3 ± 0.6	0.1 ± 0.2
Pseudomonas	"	ND	1.1 ± 1.5	2 ± 2	4.5 ± 7.5
Serratia	"	77.5 ± 20.6	76.3 ± 23.2	46.6 ±	46.5 ± 21.3
Stenotrophomonas	"	0.9 ± 1.5	1.6 ± 2.8	0.3 ± 0.4	0.1 ± 0.2
Others	"	4.4 ± 3.6	9.1 ± 1.6	0.2 ± 0.2	2.9 ± 0.5

[a] BF mosquitoes fed on naïve (uninfected) mouse; [b] Mosquitoes infected with *P. berghei* (Pb); [c]ND: not detected. The values represent the median ± SD of three independent experiments. All the bacteria that were not identified and/or resulted in a relative abundance lower than 0.3% were labelled "Other".
